# Deepening the decisional processes under value-based conditions in patients affected by Parkinson’s disease: A comparative study

**DOI:** 10.3758/s13415-024-01211-x

**Published:** 2024-09-12

**Authors:** Laura Colautti, Paola Iannello, Maria Caterina Silveri, Anna Rita Giovagnoli, Antonio Emanuele Elia, Fulvio Pepe, Eugenio Magni, Alessandro Antonietti

**Affiliations:** 1 Department of Psychology, Università Cattolica del Sacro Cuore, Largo A. Gemelli, 1, 20123, Milan, Italy; 2 Department of Diagnostics and Technology, Fondazione IRCCS Istituto Neurologico Carlo Besta, Milan, Italy; 3 Parkinson and Movement Disorders Unit, Fondazione IRCCS Istituto Neurologico Carlo Besta, Milan, Italy; 4 Department of Neuroscience, Fondazione Poliambulanza Istituto Ospedaliero Brescia, Milan, Italy

**Keywords:** Parkinson’s disease, Iowa Gambling Task, Game of Dice Task, Decision making, Executive functions, Dopamine

## Abstract

Patients affected by Parkinson’s disease (PD) display a tendency toward making risky choices in value-based conditions. Possible causes may encompass the pathophysiologic characteristics of PD that affect neural structures pivotal for decision making (DM) and the dopaminergic medications that may bias choices. Nevertheless, excluding patients with concurrent impulse control disorders, results are few and mixed. Conversely, other factors, such as individual differences (e.g., emotional state, impulsivity, consideration for future consequences) and cognitive functioning, in particular executive functions (EFs), are involved, even though few studies investigated their possible role. The present study investigated (1) the differences in value-based DM between 33 patients with PD without impulse control disorders and 33 matched healthy controls, and (2) the relationships among decisional performances, EFs, and individual differences in a group of 42 patients with PD who regularly undertake dopaminergic medications. All participants underwent an individual assessment to investigate value-based DM, cognitive abilities, and individual differences associated with DM. Nonparametric analyses showed the presence of riskier decisions in patients compared with healthy controls, depending on the characteristics of the decisional situation. Moreover, parameters of the decisional tasks involving the number of risky choices were significantly related to the posology of dopaminergic medications, EFs, and individual differences. Findings were discussed, highlighting possible clinical implications.

**Supplementary information:**

The online version contains supplementary material available at 10.3758/s13415-024-01211-x.

## Introduction

Parkinson’s disease (PD) is the second most common neurodegenerative disease, mainly caused by the depletion of dopaminergic neurons in the substantia nigra pars compacta (leading to a decrease in striatal dopamine levels) and the buildup of the α-synuclein protein forming insoluble aggregates that constitute the basis of Lewy bodies (Balestrino et al. 2020). The progression of the disease entails the involvement of corticostriatal pathways and cortical regions (Braak & Del Tredici, 2008; Braak et al., 2003).

From the early stages of the disease, in addition to the motor symptoms, it is possible to detect a wide spectrum of nonmotor symptoms, including cognitive difficulties. Among them, well-known impairments affect visuospatial abilities, attentive and executive functions (EFs) (e.g., divided attention, cognitive flexibility, inhibition, working memory, planning, executive control), speed processing, and learning (Jankovic et al., 2021). More recently, literature highlighted that patients who regularly undertake dopaminergic medications can display a tendency toward making risky and reckless choices in value-based decision making (DM), probably underlying selective impairments characterized by an insensitiveness to negative consequences from choices they made and/or an impairment in anticipating the unrewarding consequences and, consequently, in difficulties in learning from previous negative outcomes occurring in similar conditions (Cools et al., 2022; Ryterska et al., 2014).

Value-based DM is common in everyday life. It occurs when the choice of one option among several alternatives is based on the subjective value assigned to them (requiring mental operations, such as evaluating pros and cons for each option, also considering possible previous feedback and anticipating the future positive consequences (rewards) or negative ones (losses) of possible choices) (Rangel et al., 2008).

Regions belonging to the prefrontal cortex—such as the anterior cingulate cortex (ACC), the orbitofrontal cortex (OFC), and the dorsolateral prefrontal cortex (dlPFC)—and basal ganglia (BG) are deeply involved in different aspects peculiar to value-based decisions. For instance, the ACC is important for motivation, encoding choice value, error monitoring, and conflict detection. As the OFC, evidence supports that it is crucial in encoding the value of a stimulus based on previous experiences, updating stimulus-reward associations (important for reversal learning), and processing emotionally charged events (for more details, see Chau et al., 2018). The dlPFC supports operations that require high-order cognitive abilities, such as strategic planning, updating processes, and other EFs. The BG, a complex network of excitatory and inhibitory neurons, and in particular striatum, are assumed to support reward processing, learning from feedback, prediction of rewards, and behaviors motivated by rewards (Foerde & Shohamy, 2011). In this way, reinforcement learning is rooted in communication between midbrain dopamine neurons and striatum (Maia & Frank, 2011). Such structures represent key points for the nonmotor corticostriatal circuits, which are three circuits assumed to be pivotal for motivation and cognitive processes, characterized by partially overlapping corticostriatal inputs originated by distinct prefrontal areas, projecting to specific striatal regions, and remaining segregated in the BG and thalamus, and then going back to the specific cortical area (for more details, see Alexander et al., 1986; Zgaljardic et al., 2006). Specifically, the ACC, which presents connections with other subcortical regions, such as the amygdala, belongs to the anterior cingulate circuit. The OFC, which presents connections with the hypothalamus, hippocampus, and amygdala, belongs to the (lateral) orbitofrontal circuit. The dlPFC belongs to the dorsolateral prefrontal circuit.

It is claimed that the pathophysiological characteristics of the disease and the variation of dopamine levels due to the disease, together with the dopamine replacement therapy, play a role in patients’ tendency toward risky decisions, leading to changes in dopamine levels and affecting neural structures crucial for value-based DM associated to nonmotor corticostriatal circuits (Freels et al., (Freels et al. 2020); Kjær et al., 2018; Pignatelli & Bonci, 2015; Ryterska et al., 2014). In this way, according to neurocomputational models focusing on BG functioning and considering the two pathways of BG that link the striatum to PFC within corticostriatal circuits, namely, the direct excitatory pathway and the indirect inhibitory pathway, dopamine modulates reward processing through them playing a crucial role in reinforce learning (Frank et al., 2004). Phasic dopamine bursts are induced by rewards (outcomes better than expected), activating the direct pathway of the BG—that is assumed to underlie learning from positive feedback—easing a cortical response. When the next time the presentation of the same stimulus occurs and the corresponding cortical response is represented, the likelihood that such a rewarding response will be emitted is increased (sustaining reward learning) (Wiecki & Frank, 2010). While dopamine dips are elicited by outcomes worse than expected, arousing the indirect pathway that inhibits cortical responses (decreasing the likelihood to repeat the behavior through learning from punishment or negative feedback) (Wiecki & Frank, 2010; Verharen et al., 2019; Cools et al., 2022). Therefore, a lack of dopamine, as it occurs in PD, may elicit the indirect pathway activity, increasing punishment learning. Conversely, to explain the insensitiveness to negative consequences displayed by patients who regularly undertake dopaminergic medications, it is hypothesized that a contribution may be provided by taking dopaminergic medications, where exogenous dopamine may exert an overactivation of the direct pathway and concurrently an inhibition of the indirect pathway raising dopamine levels in the striatum (and preventing dopamine dips; Poletti & Bonuccelli, 2013), thus leading to learning from rewards rather than from punishment (Argyelan et al., 2018; Cools et al., 2022); Wiecki & Frank, 2010).

Likewise, the tendency toward risky choices and the insensitiveness to unrewarding consequences can be explained by mechanisms focusing on the possible effects linked to dopamine on the ventral striatum. Focusing on nonmotor circuits, throughout the earlier stages of PD, the depletion of dopamine mainly affects the dorsolateral corticostriatal circuit, leading to possible impairment in EFs, such as working memory and set-shifting, while it relatively spares the orbital circuit (mainly underlying reward processing, emotion-based representations, and reversal learning), which is usually affected in later stages (Cools et al., 2022; Poletti & Bonuccelli, 2013). Thus, the administration of dopaminergic drugs may produce differential cognitive effects on these corticostriatal circuits, sustaining those cognitive functions (such as EFs) that mainly rely on the dorsolateral circuit, but impairing those (such as value-based processes) related to the more spared ventral striatum and orbital circuit by “overdosing” them (see the dopamine overdose hypothesis: Gotham et al., 1986; Cools et al., 2022).

To delve into DM impairments in patients who regularly undertake dopaminergic medications, most studies investigated decisional performances through laboratory tasks in which assigning subjective values to options, predicting and processing rewards and losses, and learning from feedback are pivotal for making optimal choices. Studies that explored dopaminergic drug withdrawal by using these tasks (comparing the DM performance of patients when they were tested in “on” versus “off” dopaminergic medication conditions) showed an attenuated sensitivity toward punishment in pharmacological “on” conditions compared with “off” conditions and a decreased neural response to negative feedback as well (Argyelan et al., 2018; McCoy et al., 2019). Consequently, during the “on” condition, the occurrence of a greater processing of positive feedback (or reward) may explain both the tendency of patients to be more focused on reward (regardless of possible higher losses) than punishment and the impulsivity displayed by patients in making reckless choices, even in everyday life. The imbalance in learning from reward and punishment and the higher sensitivity to rewards can lead patients to develop impulse control disorders (ICDs). These are a set of behavioral disturbances characterized by a lack of voluntary control over pleasant behaviors performed compulsively, excessively, and repetitively (i.e., pathological gambling, hypersexuality, compulsive shopping, binge eating, punding, dopamine dysregulation syndrome, and hobbyism) (Gatto & Aldinio, 2019; Weintraub et al., 2015). PD patients with ICDs were estimated to range from 20 to 45% (Monaco et al., 2018; Weintraub & Claassen, 2017). In most cases, these can be side effects of the dopaminergic drug therapy (Jones et al., 2020). Accordingly, studies that investigated performances between groups of patients with PD who have ICDs versus patients with PD without ICDs showed that the former group, compared with the second or healthy controls, displayed a marked preference for choosing risky options characterized by possible immediate high rewards but also long-term higher losses (Pineau et al., 2016; Rossi et al., 2010). Conversely, results are few and mixed when behavioral performances to value-based decisional tasks of medicated patients without ICDs are considered and compared with healthy controls. Nevertheless, thorough analyses of these patients’ decisional strategies revealed the presence of a tendency toward risk as well, gaining a lower amount of money at the end of the task (when the task involved fictitious monetary wins and losses as feedback), choosing more often disadvantageous options at the end of laboratory tasks and considering negative feedback derived from previous choices to a lesser extent than the healthy control group (Colautti et al., 2021, 2023); Cools et al., 2022)for reviews). Moreover, focusing on such a population, results are inconsistent when relationships between dopaminergic medications and patients’ behavioral responses to decisional tasks are analyzed (Colautti et al., 2021; Evens et al., 2016). It remains unclear to which extent different dopaminergic medications can play distinct roles in DM (Kjær et al., 2018), because most studies considered the total levodopa equivalent daily dose (LEDD). It is possible that medications, such as dopamine agonists and levodopa, exert different effects on cognition, but these differences may be obscured by the combined total LEDD (Evens et al., 2016). Thus, it appears important to deepen value-based decisional mechanisms in patients without ICDs, which can be considered the most common clinical condition among patients with PD.

Additionally, it is worth considering that DM is a complex process in which other variables can influence the choice of an option over another one, among which emotional and behavioral states, as well as cognitive functioning of the decision-maker, play a pivotal role (Finucane & Lees, 2005); Iannello & Colautti, 2023). Focusing on cognitive functioning, a recent review shows that EFs can be a resource to promote safer and more optimal decisions under value-based conditions, especially when the cognitive demand required by the decisional situation is high (for more details, see Colautti et al., 2023a). In fact, to perform an optimal DM process, the decision-maker has to inhibit impulsive responses and consider possible long-term consequences, plan a decisional strategy, be flexible in processing available data, update possible new information, adjust future decisions based on previous feedback, and strategically recall similar previous situations (Antonietti et al., 2023; Colautti et al., 2022; Schiebener & Brand, 2015). All these abilities are encompassed in EFs. Nevertheless, results are mixed when investigating possible relationships between DM tasks and EFs, as manifold tasks have been adopted to investigate DM, underlying in different ways the main mental operations required to make a choice. Moreover, focusing on the most used tasks, only a few studies investigated whether and how the specific abilities included in the EFs can contribute to optimal decisions (Colautti et al., 2023a). A further aspect to consider is that two parameters are essential in value-based DM: the entity of positive/negative outcomes and the probability of occurrence of these outcomes (Cokely & Kelley, 2009).

The main purpose of the present study was to deepen whether and how patients with PD without ICDs are more prone to make risky choices than healthy controls (HCs) by (1) adopting the most used tasks assessing value-based DM and (2) preliminary investigating the impact of the two main parameters considered in making the choice, i.e., the entity of the positive/negative outcomes and the probability of such occurrences. So far, there is a lack of studies examining whether risk-taking behavior varies according to modifications in these parameters.

Other secondary goals were to deepen (3) the possible role played by the different types of dopaminergic medications in DM, (4) which and how specific EF abilities support DM processes in patients, and (5) whether possible emotional and behavioral differences related to DM can be related to an optimal decisional process. Because there is a lack of studies deepening the decisional mechanisms under value-based conditions in patients with PD without ICDs—even if they deserve attention as evidence in literature highlighted possible decisional impairments also in such patients—in this study we decided to focus only on patients without ICDs trying to fill the gap.

## Materials and methods

### Participants

**Fig. 1:**
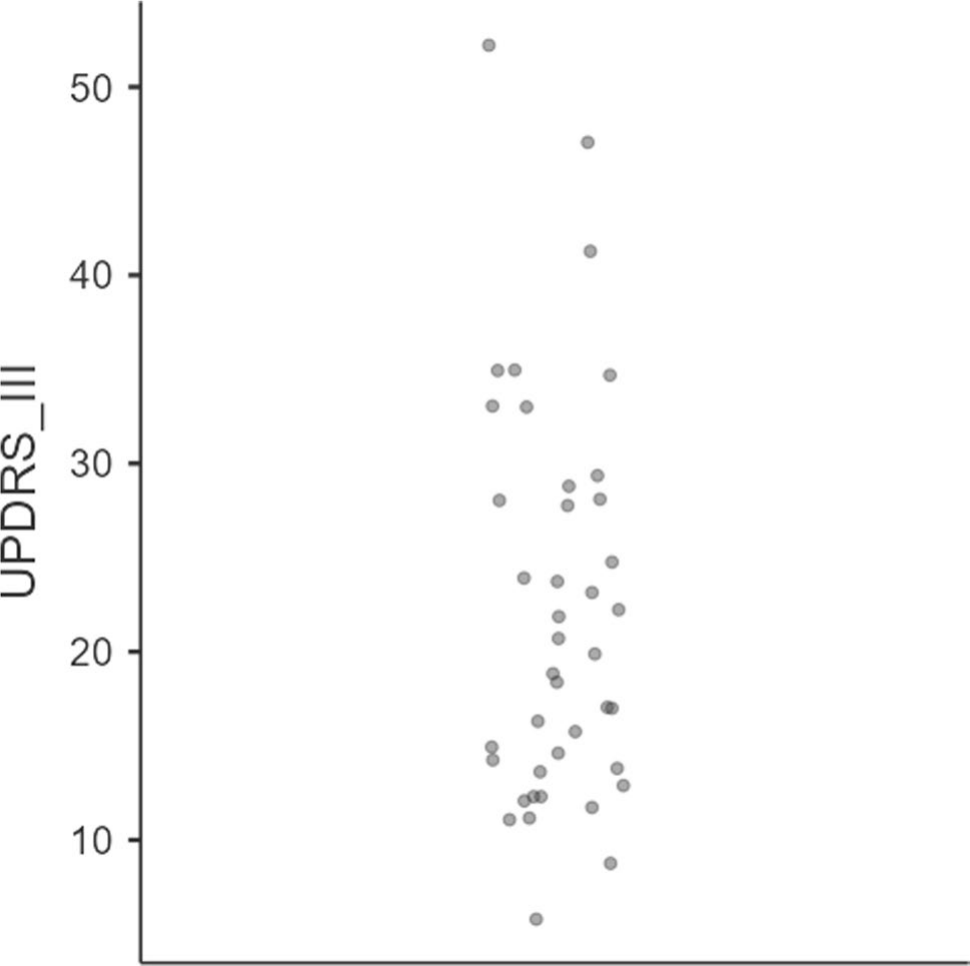
Individual data points for the severity of disease assessed through the UPDRS III (N = 42)

Forty-six patients with PD were enrolled in the study. None of them presented deficits that potentially interdict the execution of the assessment battery (e.g., severe dysarthria, severe bradykinesia, or other symptoms that could compromise the execution of the assessment). Four of them were excluded, because they did not match the inclusion criteria (see below). A total of 42 patients (52.4% male) were included (Table 1). Figure 1 illustrates patients’ severity of the disease, assessed through the Unified Parkinson's Disease Rating Scale (UPDRS) Part III (Fahn, 1987) during the pharmacological “on” condition.

**Table 1:** Characteristics of the patients with PD (N = 42)

	M	SD
Age	66.4	7.79
Years of education	9.90	3.67
MMSE	29	1.18
Disease duration	5.71	4.30
QUIP-RS total	2.52	3.28
Average year of onset	60.8	7.22
UPDRS III	22.1	10.5
LEDD (mg)		
Levodopa	290	225
DA	130	138
MAO-B inhibitors	56.2	48.2
COMT inhibitors	21.1	80.3
Total	492	308

*DA* dopamine agonists, *LEDD* levodopa equivalent daily dose, *MAO-B* monoamine oxidase-B, *MMSE* Mini Mental State Examination, *QUIP-RS* Questionnaire for Impulsive-Compulsive Disorders in Parkinson’s Disease – Rating Scale, *UPDRS III* Unified Parkinson's Disease Rating Scale – motor evaluation

Patients with Parkinson disease regularly assumed the daily dopaminergic therapy (consisting of levodopa and/or dopamine receptor agonists and/or monoamine oxidase type B (MAO-B) inhibitors. Three patients also assumed Catechol-*O*-methyl transferase (COMT) inhibitors. Four patients were taking anticholinergic drugs together with dopaminergic therapy. All patients were tested during the pharmacological “on” phase. Among the patients’ group, 33 patients (age 66.21 ± 7.64 years; education 10.61 ± 3.80 years) were matched for gender, age, and educational level to HCs (age 65.94 ± 7.77 years; education 10.82 ± 3.69 years) (Table 2 provides more details).

**Table 2:** Characteristics of the patients with PD and the matched healthy controls

	Patients n = 33	HCs n = 33	Mann–Whitney *U* test	*p*
**Gender**				
Male	14	14		
Female	19	19		
Age	66.21 ± 7.64	65.94 ± 7.77	534	.893
Years of education	10.61 ± 3.80	10.82 ± 3.69	512	.673
Neuropsychological tests				
MMSE	28.99 ± 1.08	29.10 ± 1.10	505	.599
Stroop_T	19.85 ± 12.18	15.21 ± 7.40	376	.046*
Stroop_E	1.59 ± 3.87	0.18 ± 0.55	395	.024*
FF	38.40 ± 9.75	38.99 ± 10.37	532	.873
FS	45.06 ± 6.88	49.78 ± 8.58	381	.036*
FA	33.42 ± 7.12	38.30 ± 9.02	368	.024*
SI	0.82 ± 0.18	0.87 ± 0.17	431	.145
DF	5.81 ± 0.82	6.02 ± 0.98	497	.546
DB	4.22 ± 1.15	4.50 ± 0.91	491	.496
Emotional and behavioral differences				
DASS_depression	3.57 ± 3.55	3.44 ± 3.69	421	.686
DASS_anxiety	4.73 ± 2.91	1.88 ± 2.75	165	< .001***
DASS_stress	5.30 ± 3.01	5.00 ± 3.43	495	1.00
DASS_total	13.14 ± 8.33	10.28 ± 8.42	365	.221
DII_FI	2.57 ± 1.59	4.36 ± 2.41	280	.003**
DII_DI	1.13 ± 1.31	2.09 ± 1.97	364	.061
CFC_imm	19.47 ± 9.96	25.36 ± 9.58	312	.012*
CFC_fut	25.90 ± 9.57	31.03 ± 9.30	357	.057
LCB_tot	27.57 ± 9.44	23.42 ± 10.91	381	.118
TAS_F1	15.30 ± 5.86	12.88 ± 5.25	374	.094
TAS_F2	12.52 ± 4.29	12.85 ± 4.09	465	.848
TAS_F3	20.13 ± 3.68	19.24 ± 4.70	458	.609
TAS_tot	48.28 ± 10.67	44.97 ± 11.45	404	.296

*CFC_fut* Consideration of Future Consequences Scale – future, *CFC_imm* Consideration of Future Consequences Scale – immediate, *DASS_anxiety* Depression Anxiety Stress Scale – anxiety, *DASS_depression* Depression Anxiety Stress Scale – depression, *DASS_stress* Depression Anxiety Stress Scale – stress, *DASS_tot* Depression Anxiety Stress Scale – total score, *DF* digit span forward, *DB* digit span backward, *DII_DI* Dickman Impulsivity Inventory – dysfunctional impulsivity, *DII_FI* Dickman Impulsivity Inventory – functional impulsivity, *FA* alternate fluencies, *FF* phonemic fluencies, *FS* semantic fluencies, *LCB_tot* locus of control of behavior, *MMSE* Mini Mental State Examination, *SI* shifting index, *Stroop_E* Stroop test errors, *Stroop_T* Stroop test time, *TAS_F1* Toronto Alexithymia scale – difficulty in identifying feelings, *TAS_F2* Toronto Alexithymia scale – difficulty in describing feelings, *TAS_F3* Toronto Alexithymia scale – cognitive style externally oriented, *TAS_tot* Toronto Alexithymia scale – total score

^*^p < .05; **p < .01; ***p < .001

### Procedure

Data were collected between October 2022 and November 2023. Patients affected by idiopathic PD were diagnosed by neurologists with expertise in movement disorders and were recruited from two neurological centers: Istituto Neurologico Carlo Besta in Milan and Fondazione Poliambulanza Istituto Ospedaliero in Brescia. Both centers are located in Lombardy, a region of Northern Italy.

Inclusion criteria were (1) the presence of a diagnosis of idiopathic PD according to the UK Parkinson’s Disease Society Brain Bank diagnostic criteria (Clarke et al., 2016) for at least 1 year; (2) unimpaired global cognitive functioning: Mini Mental State Examination (MMSE; Measso et al., 1993) ≥ 24; UPDRS Part I item 1 ≤ 1; (3) age 50 to 80 years; (4) a stable dopaminergic therapy for at least 2 months; (5) the absence of severe motor or nonmotor fluctuations; (6) the absence of severe ICDs; and (7) psychological profile in the normal range or characterized only by mild symptoms of depression or anxiety.

Exclusion criteria were (1) refusal to sign the informed consent; (2) the presence of neurologic, severe systemic, or psychiatric comorbidity; (3) the presence of progressive or severe brain injury documented by nuclear magnetic resonance imaging (MRI); (4) the presence of deep brain stimulation or other previous neurosurgical interventions; and (5) the presence or a history of severe addictions or any ICDs.

First, all the recruited patients underwent a neurological examination. Subsequently, for each patient, two individual in-presence sessions—each lasting approximately 45 min—were scheduled. During the sessions, an assessment battery was administered, including neuropsychological tests and decisional tasks. Tests and tasks were performed in random order, except for MMSE and Questionnaire for Impulsive-Compulsive Disorders in Parkinson’s Disease–Rating Scale (QUIP-RS; Weintraub et al., 2012), which were always proposed as the first tools to provide data for inclusion criteria.

Information about clinical history (e.g., onset age of the disease, year of the diagnosis, drugs taken, possible comorbidities) was collected before the assessment for both descriptive purposes and to verify the satisfaction of the inclusion criteria.

The LEDDs—divided according to each type of medication (levodopa, dopamine agonists, MAO-B inhibitors, and COMT inhibitors)—were considered, calculated according to Tomlinson and colleagues ( 2010). Schade and colleagues’ (2020) study was considered for newer medications (such as opicapone, safinamide, and extended-release levodopa).

Moreover, a sample composed of HC participants matched to patients for gender, age, and education level was recruited mainly from sociocultural centers, soft gymnastics gyms, and neighborhood stores in Lombardy. Inclusion criteria were (1) MMSE ≥ 24 and (2) the absence of a history of neurologic, severe systemic, or psychiatric disorders. Exclusion criteria were (1) refusal to sign the Informed Consent; (2) previous neurosurgical interventions; and (3) the presence or a history of severe addictions.

No incentive was given to participate in the study. Written informed consents were collected from all patients with PD and HC participants before starting the individual sessions. The study was approved by the Ethic Committee of the recruited centers: Istituto Neurologico Carlo Besta (no. MAP_DEC), Fondazione Poliambulanza Istituto Ospedaliero (no. PD_DEC), and Università Cattolica del Sacro Cuore (approval code 96–21). The study was conducted according to the standards of the Helsinki Declaration (World Medical Association, 2013).

To compute the a priori required sample size, the Game of Dice Task (GDT; Brand et al., 2005) was used, as one of the most used tasks. Mean GDT scores (final outcome) were considered. Specifically, the literature (Brand et al., 2004); Euteneuer et al., 2009) suggests, considering the comparison between patients with PD and HCs in DM assessed by the score (final outcome) at the GDT, that mean GDT scores (final outcome) are approximately − 2500/ − 3000 in patients with PD and approximately − 600/700 in controls. In detail, the following mean and standard deviation values for GDT were observed in Euteneuer and colleagues’ (2009) study: mean = 2654.14 (SD = 3283.07) for PDs and mean =  − 786.96 (SD = 1663.87) for controls. With these values, an effect size from the difference in mean of 0.72 is obtained. Using a two-way Mann–Whitney *U* test (power 0.80, alpha error 0.05), the minimum effect size for this effect to show a statistically significant difference between groups (with 1:1 allocation) is N = 66 (n1 = n2 = 33 per group).

### Materials

A neuropsychological battery was designed to assess cognitive functioning and DM under uncertain and risky conditions, which are two common value-based conditions.

#### Decisional tasks

- Iowa Gambling Task (IGT; Bechara et al., 1994; Mueller & Piper, 2014) was employed to assess DM under uncertainty, a common value-based condition. It is a computerized task, based on the original version of Bechara and colleagues (1994), in which the subject has to increase as much as possible an initial sum of money ($2000) by selecting cards from four decks (100 trial choices are scheduled). Two decks are safe (or advantageous, i.e., C and D), because they, if chosen repeatedly over time, lead to a total gain higher than the losses. The other two decks are risky (or disadvantageous, i.e., A and B), because they, if chosen repeatedly over time, lead to consistent losses. The two safe decks differ from each other in the frequency and the entity of gains and losses, and the same is true for the risky ones. Participants were not informed about the features of the decks. Thus, to optimize the outcome, the participant has to understand, based on feedback from previous choices, which are the advantageous decks. The considered parameters are the netscore (advantageous minus disadvantageous selections) in the whole game and in each of the five blocks (of 20 trials each) in which the task can be divided and the total amount of money at the end of the game.
- Game of Dice Task (GDT; Brand et al., 2005) was used to assess DM under risk, another common value-based condition. It is a computerized task, in which the subject has to bet which face/number will come out at each roll of a die (for a total of 18 rolls), with the goal to increase an initial amount of money. For the choice related to each roll, both the probabilities and the specific wins and losses about possible choices have been explicitly expressed. In fact, the player can choose a single number, receiving the highest monetary win if it occurs (1:6 chance) or losing the highest one (5:6,), or can choose a combination of two numbers, with a lower winning value but a greater chance to win (2:6 chance), etc. for the combination of three and four numbers. The considered parameters are the netscore (safe (three and four faces of the die) minus risky (one or two faces of the die) choices) and the total sum of money earned.
- Drawn Lots Task (DLT; Colautti et al., 2023b), for more details see https://osf.io/cpjh4/?view_only=23bef8d21ee84b78a35d416e80803437) was conceived to investigate the weight of the entity and the probability of occurrence in the decisional process. The task consists of 30 trials, equally divided into three parts. In each trial, two boxes are proposed between which the subject has to choose the most advantageous one, with the goal to increase the total gain as much as possible at the end of the task. Each part has peculiar characteristics. Specifically, in the first part, odds or probabilities of winning/losing for each box are declared, but only the rough range of the entity of gains and losses is declared. In the second part, the value or entity of winning/losing for each box is specified, but only the rough range of the probabilities of win or lose is declared. In the third part, both the probability and the entity of winning/losing are given. The boxes in each pair have the same expected value. No trial has a 100% probability of winning. For each part of the task, the probability-entity associations are kept the same. For instance, in part 1, trial 3, box A presents a 70% of chance of winning and a 30% of chance of losing from 5 to 25 euros. In part 2, trial 3, box A presents the gain or loss of 15 euros, with an explicit range of probability of occurrence of the gain within the 60% to 80% interval. In part 3, trial 3, box A is presented with a 70% of chance of winning 15 euros and a 30% of chance of losing 15 euros. The same mechanism is true for the other pairs of boxes. This is intended to limit possible biases due to not controlled characteristics of the boxes across the three parts. At the end of each part, feedback is provided to the subject regarding the total gain (or loss) derived from the choices taken. Wins and losses are counted according to a fixed outcome derived from a draw conducted during the task-design phase for each box. To avoid possible biases due to the order of presentation of the three parts, three orders of presentation were devised to counterbalance them among participants (first order: part 1 – 2 – 3; second order: part 2 – 3 – 1; third order: part 3 – 1 – 2), randomly assigned to participants so each sequence was assigned approximately to the same number of participants. 
  The instructions given to the subjects were: “On each sheet of paper there are two boxes (A and B), each containing green and orange balls. The extraction of a green ball produces a win, while the extraction of an orange ball produces a loss. In each box the number of green and orange balls is different and, so, the chance of winning and losing is different as well. The entity of the win and loss also is different from one box to another. In each sheet, you have to choose one of the two boxes. As soon as you select the box, automatic random extraction of a ball will take place. Your goal in this game is to earn as much money as possible and lose as little as possible.” 
  The considered measures for each part of the task is the number of safe choices. Moreover, at the end of the task, the participant was asked which part of the task has been perceived as the most difficult for making a choice.

#### Neuropsychological tests

- Mini Mental State Examination (MMSE; Measso et al., 1993) to assess global cognitive functioning and verify one of the inclusion criteria. The test is composed of 30 items that investigate seven different cognitive domains (temporal and spatial orientation, words registration, attention and calculation, words recall, language, and constructive praxis). The total score ranged between a minimum of 0 and a maximum of 30 points (one point for each correct answer).
- Stroop test – short version (Caffarra et al., 2002) was administered to assess the inhibitory self-control ability in the presence of verbal interferences. The test consists of three parts: (1) the subject has to read words of colors (“blue,” “green,” “red”); (2) he or she has to name the color of some dots (colored blue, green, or red); (3) the subject has to name the color of the ink (colored blue, green, or red) of some words of colors (“blue,” “green,” “red”). The time to complete the three parts of the task is recorded. Two parameters were computed: “Time” (the longer the subject takes to perform the task, the more the subject is affected by the cognitive interference) and “Errors” (the greater the number of errors during the performance, the more the subject has difficulty in controlling the interference).
- Alternate fluencies (Costa et al., 2014) was aimed to assess set-shifting and verbal strategic flexibility through tests of lexical access. It is composed of three parts: (1) Phonemic fluency, in which the subject has to say as many words as possible beginning with a letter within 60 s. Three trials compose this part (letters are A, F, and S); (2) Semantic fluency, in which he/she has to generate as many words as possible belonging to certain categories within 60 s (three trials compose this part, each corresponding to a category: colors, animals, and fruits); (3) Alternate phonemic/semantic fluency, in which the subject has to alternate words beginning with a declared letter with words belonging to a category within 60 s (three trials compose this part: A-colors, F-animals, and S-fruits); There is a score for each part, based on the number of correct words generated. Moreover, considering all the pronounced words in the three parts, a fourth score is computed, called “Shifting index,” which detects an extradimensional set-shifting skill, reflecting how much the subject is able to move from one mental set to another.
- Digit Span Backward (DB; Monaco et al., 2013) assesses working memory (or updating ability), namely, the ability to keep information in memory performing goal-oriented cognitive processing. The subject has to repeat a sequence of digits, gradually increasing in length, in reverse order. The score corresponds to the length (i.e., the number of digits) of the longest sequence correctly recalled.
- Digit Span Forward (DF; Monaco et al., 2013) evaluates short-term memory. The subject has to repeat a sequence of digits, gradually increasing in length, in forward order. The score corresponds to the length (i.e., the number of digits) of the longest sequence correctly recalled. This test has been inserted in the cognitive evaluation to reduce the possibility of including participants with possible short-memory impairments in the sample.

#### Impulse control disorders

- Questionnaire for Impulsive-Compulsive Disorders in Parkinson’s Disease – Rating Scale (QUIP-RS; Weintraub et al., 2012) was proposed to patients with PD during the anamnestic interview to exclude the presence of severe ICDs. The QUIP-RS is a 5-point Likert scale (ranging from 0 = not at all, to 4 = very often) and measures the presence and the severity of impulse control behaviors by asking the frequency of seven main behavioral disorders over the past 4 weeks. The behavioral disorders are gambling, compulsive shopping, hypersexuality, compulsive eating, hobbyism, iteration of simple activities/punding, and dopamine dysregulation. They are investigated through four questions that explore four main aspects of the ICD: (1) ideation oriented toward the behavioral disorder; (2) desire and drive toward the pathological behavior; (3) inability to inhibit the pathological behavior; and (4) implementation of abnormal actions to realize the compulsive behavior. Normative data are provided to identify threshold scores above which each of the main behavioral disorders should be considered clinically relevant (gambling ≥ 6, compulsive shopping ≥ 8, hypersexuality ≥ 8, compulsive eating ≥ 7, hobbyism ≥ 7, iteration of simple activities/punding ≥ 7, except for dopamine dysregulation behavior, of which none of the patients within the normative sample displayed such behavior; Weintraub et al., 2012). Scores were used to screen the possible presence of ICDs (which was an exclusion criterion).

Because emotional and behavioral differences also can influence value-based DM (Poletti et al., 2011; Zhang et al., 2015), self-report scales were proposed:

- Depression Anxiety Stress Scale (DASS-21; Bottesi et al., 2015) is composed of 21 items (representing 21 symptoms) on a 4-point Likert scale (ranging from 0 = It has never happened to me, to 3 = It happened to me most of the time). It is required to answer by referring to the last week. By summing the respective answers, three scores can be derived to assess depressive symptoms, anxiety, and stress levels. By summing all the answers, it is possible to get a score indicating the general level of distress (depression: McDonald’s ω = 0.869; anxiety: McDonald’s ω = 0.763; stress: McDonald’s ω = 0.812; total: McDonald’s ω = 0.921).
- Dickman Impulsivity Inventory (DII; Colledani, 2018; Dickman, 1990) is composed of 18 items (answers are true or false). The tool is divided into two subscales that investigate functional impulsivity (9 items), which refers to the tendency to act quickly when this style is appropriate, and dysfunctional impulsivity (9 items), which refers to behaving quickly and defectively in a way that has unfavorable repercussions (functional: McDonald’s ω = 0.712; dysfunctional: McDonald’s ω = 0.699).
- Locus of Control of Behavior (LCB; Farma & Cortivonis, 2000) is composed of 17 items on a 6-point Likert scale (ranging from 0 = Completely disagree, to 6 = Completely agree). It investigates the “locus of control” (internal/external) that the respondent usually adopts in various situations. The overall score is the sum of the responses, where the higher the score is, the higher is the external locus of control (McDonald’s ω = 0.775).
- Consideration of Future Consequences-14 Scale (CFC-14; Nigro et al., 2016) is composed of 14 items on a 7-point Likert scale (ranging from 1 = It does not represent me at all, to 7 = It totally represents me). The tool is divided into two subscales investigating CFC-Immediate, which refers to the preference to consider immediate rewards rather than long-term consequences of a choice, and CFC-Future, which indicates the ability to consider long-term consequences of a possible choice (immediate: McDonald’s ω = 0.871, future: McDonald’s ω = 0.832).
- Toronto Alexithymia Scale (TAS-20; Bagby et al., 1994) is composed of 20 items on a 5-point Likert scale (ranging from 1 = Completely disagree, to 5 = Completely agree). It investigates the presence of alexithymic traits, related to the presence of alterations in emotional regulation (Taylor et al., 1991). It is composed of three main dimensions concerning the difficulty in identifying feelings (F1 subscale), the difficulty in describing feelings (F2 subscale), and a cognitive style externally oriented (F3 subscale). By summing all items, a total score can be considered (F1: McDonald’s ω = 0.838; F2: McDonald’s ω = 0.704; F3: McDonald’s ω = 0.445; total: McDonald’s ω = 0.824).

## Results

### Statistical analyses

The statistical analyses were performed by using Jamovi (version 2.3.13). First, to check the normality of the variables, the analysis of the asymmetry and skewness and the Shapiro–Wilk test were performed, revealing that most of the variables were not normally distributed (Shapiro-Wilk: *p*< 0.05) (McKnight & Najab, 2010).

The internal consistency of each administered scale was assessed through reliability analyses adopting McDonald’s ω. The F3 subscale belonging to the TAS-20 was not considered in the analyses, because it has a value below the threshold of acceptability (Hayes & Coutts, 2020; McNeish, 2018).

Then, to analyze possible differences between patients and matched HCs, Mann-Whitney *U* test was used in relation to age, educational level (number of years), and scores in decisional tasks, cognitive tests (adopting the adjusted raw scores computed according to the correction grids reported in the tests’ validation articles), and self-report questionnaires.

To delve into possible relationships between parameters of the decisional tasks and (1) the cognitive tests, (2) the self-report questionnaires mentioned, (3) the clinical characteristics of the disease (e.g., duration of the illness, age at the onset), and (4) the dopamine replacement therapy, correlation analyses using Spearman’s ρ (Schober et al., 2018) were performed in the PD group only. When relationships between categorical variables were investigated, the χ^2^ was used.

### Comparing patients with PD and HCs

#### Demographic data and neuropsychological tests

Demographic and neuropsychological data of the PD and HC groups are shown in Table 2. The Mann-Whitney *U* test highlighted no significant differences in terms of age, years of education, and MMSE score. Conversely, the two groups differed in scores obtained in the Stroop test and semantic and alternate fluencies, revealing higher difficulties by patients with PD in inhibiting irrelevant stimuli and in cognitive flexibility. Moreover, patients with PD displayed higher levels of anxiety and lower levels of reported impulsivity (both functional and dysfunctional).

### Decision-making performances between the PD group and HC group

Table 3 summarizes the differences in DM performances for the administered tasks between patients with PD and HCs.

**Table 3:** Differences of DM performance between patients with PD and HCs (Mann–Whitney *U* test)

	Patients with PD n = 33	HC group n = 33	Mann–Whitney *U* test	*p*
IGT_totalamount	1755.71 ± 691.459	1870.65 ± 719.984	486	.453
IGT_netscore_tot	2.79 ± 20.57	7.76 ± 27.66	453	.243
IGT_netscore_1-20	− 2.06 ± 4.23	− 3.82 ± 4.73	434	.151
IGT_netscore_21-40	0.49 ± 4.12	3.15 ± 5.96	386	.040*
IGT_netscore_41-60	1.33 ± 5.91	3.33 ± 8.81	439	.176
IGT_netscore_61-80	0.79 ± 5.94	3.52 ± 9.26	414	.093
IGT_netscore_81-100	2.24 ± 7.16	1.58 ± 10.59	526	.811
A	21.76 ± 7.04	18.27 ± 6.46	387	.043*
B	26.85 ± 6.76	27.85 ± 11.30	534	.893
C	23.03 ± 5.48	23.52 ± 8.71	534	.898
D	28.36 ± 11.09	30.36 ± 8.89	426	.128
GDT_totalamount	− 1639.394 ± 2494.49	− 709.09 ± 3658.58	348	.012*
GDT_netscore	4.36 ± 9.32	5.33 ± 10.41	491	.495
GDT_risky_tot	7.33 ± 4.85	6.33 ± 5.21	463	.295
GDT_safe_tot	10.67 ± 4.85	11.67 ± 5.21	463	.295
GDT_1	3.52 ± 3.58	2.03 ± 3.64	361	.015*
GDT_4	7.73 ± 4.25	6.61 ± 5.38	444	.198
DLT_part1	7.64 ± 2.51	8.03 ± 1.90	520	.746
DLT_part2	5.06 ± 3.16	7.03 ± 2.80	450	.224
DLT_part3	7.73 ± 2.70	7.88 ± 2.32	538	.931

*DLT_part1* Drawn Lots Task – number of safe choices in the first part, *DLT_part2* Drawn Lots Task – number of safe choices in the second part, *DLT_part3* Drawn Lots Task – number of safe choices in the third part, *GDT_1* Game of Dice Task – number of time the riskiest choice was made, *GDT_4* Game of Dice Task – number of time the safest choice was made, *GDT_risky_tot* Game of Dice Task – number of risky choices, *GDT_safe_tot* Game of Dice Task – number of safe choices, *IGT* Iowa Gambling Task

^*^p < .05; **p < .01; ***p < .001

#### Iowa Gambling Task

Regarding the IGT, although the total amount gained at the end of the task was not significantly different (*U* = 486, *p* = 0.45), patients with PD gained a lower amount than the HCs. Significant differences emerged in the second block (*U* = 386, *p* = 0.04) in the number of selections of deck A (*U* = 387, *p* = 0.043), revealing that patients with PD made less advantageous choices in the second block and chose more frequently cards from deck A.

#### Game of Dice Task

In the GDT, analyses revealed a significant difference between the two groups in the total amount earned at the end of the task (*U* = 348, *p* = 0.012) and in the number of times participants chose the riskiest option (*U* = 361, *p* = 0.015), pointing out that patients with PD earned a significantly lower amount and chose more frequently the riskiest option. No other significant differences emerged.

#### Drawn Lots Task

In the DLT, no significant differences emerged in the number of safe choices for the three parts of the task. To explore possible differences between the two groups in the DLT part perceived as more difficult for making a choice, χ^2^ analysis was performed, revealing no significant differences (*p* = 0.862). Both groups perceived the second part as the most difficult to make a decision.

### Focus on patients with PD

#### Correlations between decisional tasks and the anamnestic data

The total netscore of the IGT did not present significant correlations with sociodemographic data (age, years of education) or with clinical data controlling for the age of patients (age at the onset, duration of the disease, severity of the disease). The netscore of the GDT significantly correlated only with the age (*ρ* =  − 0.365, *p* = 0.018): the more the age, the poorer the decisional performance under risky conditions.

#### Correlations between decisional tasks and dopaminergic medications

Considering the IGT, significant correlations emerged between the second block netscore and the LEDD of dopamine agonists (*ρ* =  − 0.371, *p* = 0.015; Table S1). Concerning the GDT, the LEDD of levodopa significantly correlated with the netscore, the number of risky choices (therefore, in the opposite direction with the number of safe choices), and the riskiest option (*ρ* =  − 0.407, *p* = 0.007; *ρ* = 0.385, *p* = 0.012, *ρ* = 0.323, *p* = 0.037, respectively; Table S2). The LEDDs of MAO-B inhibitors and of COMT inhibitors did not show significant correlations with either of the decisional tasks.

Thus, it emerged that the greater the daily intake of dopamine agonists, the more the number of risky choices made in the second block of the IGT. Therefore, under conditions of uncertainty, while the greater the daily intake of levodopa, the more the number of risky choices made under conditions of risk.

#### Correlations between decisional tasks and neuropsychological tests

Considering the total sample of patients with PD, analyses revealed significant correlations between the IGT netscores and the neuropsychological tests. Specifically, the IGT total netscore correlated with the alternate fluencies and the shifting index (*ρ* = 0.309, *p* = 0.047; *ρ* = 0.347, *p* = 0.024). Analyzing the IGT blocks to deepen the learning process from feedback, scores in the first block correlated with Stroop errors and phonemic fluencies (*ρ* = 0.389, *p* = 0.012; *ρ* = 0.422, *p* = 0.005, respectively), whereas the second block correlated with digit backward (*ρ* =  − 0.388, *p* = 0.011, respectively), the third with digit backward (*ρ* = − 0.326, *p* = 0.035), the fourth with the shifting index and the digit backward (*ρ* = 0.378, *p* = 0.014; *ρ* =  − 0.345, *p* = 0.025), and the fifth with digit backward (*ρ* =  − 0.369, *p* = 0.016; Table S3). Thus, under uncertain conditions, the more the flexibility, the more the advantageous choices made in general and in particular in the first and fourth block, whereas the more the working memory, the more the number of disadvantageous choices made.

Regarding the GDT, the netscore and the riskiest option significantly correlated with Stroop time (*ρ* =  − 0.371, *p* = 0.017; *ρ* = 0.393, *p* = 0.011, respectively; Table S4). Thus, under risky conditions, the more the inhibition, the less the number of risky choices made.

#### Correlations between decisional tasks and emotional and behavioral differences

Analyzing the IGT netscores, the total netscore significantly correlated with the DASS subscale of anxiety (*ρ* =  − 0.329, *p* = 0.043), the functional and dysfunctional impulsivity subscales of the DII (*ρ* = 0.397, *p* = 0.014; *ρ* =  − 0.448, *p* = 0.005, respectively), the future subscale of the CFC (*ρ* = 0.363, *p* = 0.025), and the TAS F1 subscale and total score (*ρ* =  − 0.341, *p* = 0.036; *ρ* =  − 0.328, *p* = 0.047, respectively). The second block correlated with the future subscale of CFC (*ρ* = 0.414, *p* = 0.010), the third block with the functional and dysfunctional subscales of the DII, and the future subscale of the CFC (*ρ* = 0.491, *p* = 0.002; *ρ* =  − 0.547, *p* < 0.001; *ρ* = 0.383, *p* = 0.018). The fourth block correlated with the depression and anxiety subscales of the DASS, the functional and dysfunctional subscales of the DII, the LCB, and the TAS F1 subscale and total (*ρ* =  − 0.383, *p* = 0.021; *ρ* =  − 0.424, *p* = 0.008; *ρ* = 0.392, *p* = 0.015; *ρ* =  − 0.505, *p* = 0.001; *ρ* =  − 0.387, *p* = 0.016; *ρ* =  − 0.383, *p* = 0.018; *ρ* =  − 0.379, *p* = 0.021, respectively; Table S5). Therefore, under uncertain conditions, the more the negative emotional states, such as anxiety, the adoption of an external locus of control, the attitude to act impulsively and recklessly, the more the disadvantageous choices made. Conversely, the more the attitude to consider the long-term consequences of actions and the ability to identify feelings, the more the advantageous choices made.

**Fig. 2:**
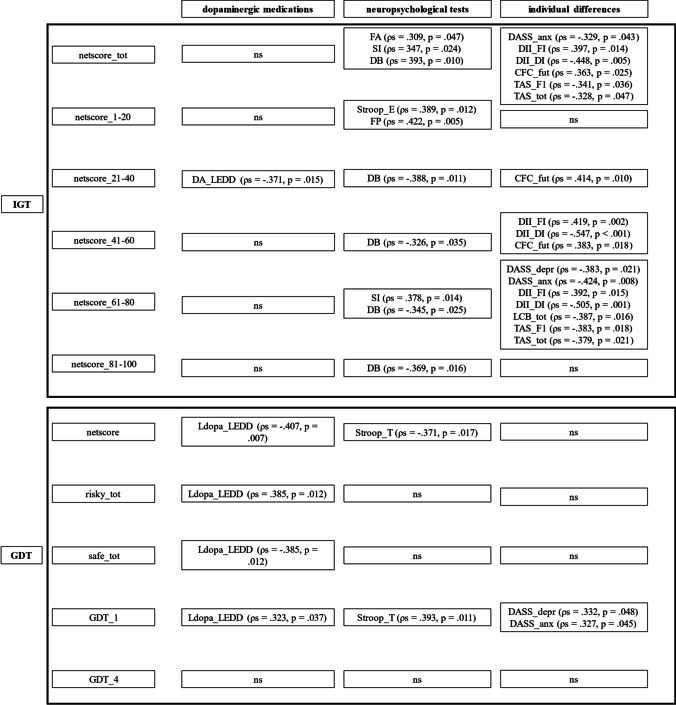
Correlations between decisional tasks (i.e., IGT and GDT), dopaminergic medications, neuropsychological tests, and emotional and behavioral differences. Note: CFC_fut = Consideration of Future Consequences Scale – future; DA_LEDD = levodopa equivalent daily dose of dopamine agonists; DASS_anxiety = Depression Anxiety Stress Scale – anxiety; DASS_depression = Depression Anxiety Stress Scale – depression; DB = digit span backward; DII_DI = Dickman Impulsivity Inventory – dysfunctional impulsivity; DII_FI = Dickman Impulsivity Inventory – functional impulsivity; FA = alternate fluencies; FP = phonemic fluencies; LCB_tot = locus of control of behavior; Ldopa_LEDD = levodopa equivalent daily dose of levodopa; SI = shifting index; Stroop_E = Stroop test errors; Stroop_T = Stroop test time; TAS_F1 = Toronto alexithymia scale – difficulty in identifying feelings; TAS_tot = Toronto alexithymia scale – total score

Concerning the GDT, significant correlations emerged only between the riskiest option and both the depression and the anxiety subscales of the DASS (*ρ* = 0.332, *p* = 0.048; *ρ* = 0.327, *p* = 0.045, respectively; Table S6). Thus, under conditions of risk, the more the negative emotional states, the more the riskiest choices made. See Fig. 2 for an overall picture of results.

## Discussion

Our first goal was to verify possible differences in DM performances by comparing a sample of patients with PD to a sample of matched HCs. In the IGT, patients with PD globally performed more poorly than HCs, showing the tendency toward disadvantageous choices, in line with reviews available in the literature (Colautti et al., 2021; Evens et al., 2016; Kjær et al., 2018). Patients chose significantly more often cards from deck A, which is characterized by higher wins but also 50% of losses occurrence (Bechara, 2007; Buelow & Suhr, 2013). Furthermore, by analyzing the netscores of each block of the IGT, both groups improved their DM performance after the first block, even if patients performed differently from HCs. While HCs began recognizing advantageous decks after the first 20 trials, outperforming patients in the second block, patients with PD showed a slower learning trend. This is in line with studies that investigated PD without ICDs analyzing the IGT blocks’ netscores, where usually HCs choose more advantageous decks than patients with PD around the second or third block (blocks in which it is assumed that individuals without cognitive impairments begin to prefer advantageous decks, starting to build a correct representation of the decks based on previous feedback; Brand et al., 2007).

In the GDT, we found that patients with PD earned a significantly lower amount than HCs and chose more frequently the riskiest option, which explicitly provides the smallest probability of winning the largest amount but also the highest probability of losing the largest amount. Such results confirm the literature supporting the presence of DM impairments under risky conditions in patients with PD (Brand et al., 2004; Euteneuer et al., 2009; Xi et al., 2015).

In the DLT, both groups encountered more difficulties in making a decision when the precise probability of the win-and-loss occurrence was not provided. Such a result supports the importance of probability weighting in making optimal choices (Bruhin et al., 2022). Although further studies are needed to confirm such results, we can suppose that conditions in which there is a lack of information about the possible consequences of a choice are more critical for the decision-maker, especially when situations are unusual. So, previous knowledge and crystallized intelligence play a minor role.

Moreover, results revealed that patients with PD made choices similar to the HCs in the three parts of the task. The absence of significant differences may be explained, at least in part, by the structure of the DLT and the feedback modality. In this task, feedback is not given after each choice (as it conversely happens in the IGT or in the GDT) but at the end of each part of the task (i.e., every ten choices). In this regard, the literature shows that patients with PD perform similarly to HCs when the DM task does not provide “discrete” feedback (namely, when the win-or-loss information is provided after each trial, as it happens in the IGT and in the GDT) and the presence of feedback is a main predictor of the presence of DM impairments in patients with PD (Ryterska et al., 2013) and can increase the tendency to make riskier choices (Labudda et al., 2010). To explain such a behavior, it is assumed that patients with PD are not globally impaired in DM, but in specific decisional steps, such as outcome evaluation and, especially, processing and using feedback (Ryterska et al., 2014). In tasks where discrete feedback occurs and provides information about the cue-outcome relationship, a pivotal role may be played by BG, in particular by the striatum and the phasic modulation of firing in dopaminergic neurons, crucial for learning to predict rewarding outcomes and taking actions to get them, and which evidence shows that can be critical in medicated patients (as reported in the Introduction section) (Foerde & Shohamy, 2011; Ryterska et al., 2013, 2014). In conditions where feedback is not provided, the involvement of such operations (namely, the representation of reward and reward prediction error signals) is lower, because it is required to choose between options without learning from feedback, and so patients with PD may rely to a lesser extent on such impaired neurocognitive mechanisms (Minati et al., 2011; Ryterska et al., 2014). Thus, these findings seem to support the assumption that BG can be crucial (and maybe selectively involved) to support learning processes that are guided by feedback and motivated by rewards. More studies are needed to better disentangle such an issue, which to date has rarely been investigated in this field.

### Focus on patients with PD

From analysis of patients with PD only, the findings can shed light on the possible mechanisms that underly value-based DM in patients with PD, considering also dopaminergic medications, EFs, and individual differences in emotion and behavior components. Consistent with the literature, DM performance was not correlated with patients’ clinical characteristics, such as the severity of the disease or its duration.

#### Relationships between DM and dopaminergic medications

Starting from the dopaminergic replacement therapy, we found a significant inverse relationship between the LEDD of dopamine agonists and the second block of the IGT netscore. It seems interesting that only one significant relationship emerged—just in that block in which there was a significant difference in the decisional performance between patients and HCs and in which it is assumed that (healthy) subjects begin to learn from trials’ feedback and build a representation of the choice options (decks). Such a result can be supported by studies showing that dopamine agonists, affecting tonic firing of dopamine cells by activating D2 dopamine receptors, can reduce the inhibitory function of the indirect pathway affecting learning from negative feedback (hindering the effects of dopamine dips) (Frank et al., 2004; Wiecki & Frank, 2010; Moustafa et al., 2012; Verharen et al., 2019). In this way, patients may develop a bias toward rewarding outcomes, but not punishments, preferring decisional strategies focused on finding wins rather than avoiding losses. Because no other significant relationship between the IGT performance and the LEDD of dopamine agonists emerged, as well as no other significant difference comparing the IGT performance between PD without ICDs and HC groups among the IGT blocks, we can hypothesize that dopamine agonists may have not impaired all decisions made in the IGT but probably may have contributed to slow down patients’ learning from negative feedback, leading them to make a higher number of risky choices.

Conversely, under the conditions of risk assessed through the GDT, where the win-and-loss probabilities are declared and remain stable over time, we found significant negative relationships between the LEDD of levodopa and both the GDT netscore and the number of safe choices (and so, in the opposite direction, the number of risky choices) and a positive relationship between the LEDD of levodopa and the riskiest choice. Such results highlight the possible presence of different cognitive mechanisms involved in the GDT and in the IGT. In the IGT, we argue that patients may present (at least in some trials of the task) a preference toward risky choices because of an insensitiveness for previous negative outcomes (whose probabilities of occurrence are never explicitly stated). In the GDT, we can suppose that patients with PD made risky choices searching the highest rewarding conditions that can derive from them, even if they knew the reduced probability of winning. Such a speculation can be supported by evidence showing differences between the effects of dopamine agonists and levodopa on the BG circuits. As previously said, dopamine agonists act on D2 receptors affecting tonic firing of dopamine cells, whereas levodopa acts on D1 and D2 receptors, affecting both phasic and tonic firing of dopamine cells (Calabresi et al., 2008). Dopamine D1 receptor signals in the ventral striatum are assumed to underlie learning from positive feedback (Verharen et al., 2019), possibly increasing sensitivity toward positive feedback, which may explain the search of options connoted by the higher wins, regardless of the high probability of high losses. Thus, in the case of the IGT, risky choices may be guided by an insensitiveness to losses, which leads to more disadvantageous choices slowing down the learning process due to negative feedback, whereas in the GDT risky choices can be led to a focus on positive feedback, possibly neglecting the negative consequences and facilitating reward-based behaviors. Two similar, but different, mechanisms can lead to the preference toward risky choices. To our knowledge, this is the first study that thoroughly investigated the relationships between the DM processes under value-based conditions and the LEDDs for each type of dopaminergic medication (while most studies in literature investigated mainly the total LEDD without finding significant results). Future studies are needed to confirm such preliminary results.

Similarly, further studies that also consider patients with PD affected by ICDs should be desirable to compare DM performances and LEDDs divided according to each type of medication in patients with and without ICDs, for a better understanding of possible differences in the neurocognitive mechanisms underlying risky choices in such patients.

#### Relationships between DM and EFs and individual differences

In the IGT, good levels of cognitive flexibility were related to the tendency to make more advantageous choices and avoid risky ones. Instead, negative relationships between the IGT netscores and working memory (assessed through the Digit span backward) were found. Such relationships with working memory (for which there were significant relationships with almost all the IGT parameters) seem counterintuitive considering the cognitive abilities involved in the IGT. Moreover, to date few studies have investigated the role played by specific EF abilities along the IGT blocks, because most of them adopted “complex tests,” such as the WCST or have analyzed only the total netscore (Colautti et al., 2023a). A possible hypothesis that can explain our results is the presence of a “fatigue effect,” which biased behavioral data, even though we can exclude it as the order of the tests was randomized. Other possible hypotheses can be formulated by keeping in mind that in PD working memory can be enhanced or impaired depending on task demands (Cools et al., 2010), as well as the basal level of dopamine, the underlain BG circuit (dorsal or ventral, where the latter is more spared from dopamine depletion in the first stages of the disease), and the evolution of the disease (Cools et al., 2003). In this way, compared with the digit span backward structure, the working memory load required by the IGT is different; in the former test, it is required to maintain active in memory only a sequence of numbers per time to reverse it, whereas in the latter task, it is required to upload the representation of each deck every time feedback occurs and to change it if no longer consistent with subsequent feedback, possibly involving to a different extent mental operations, such as selection and maintenance. Moreover, the IGT includes an affective component not involved in the digit span backward. Such an affective component can involve to a greater extent circuits related to the ventral caudate and ventral striatum than the digit span backward (which, conversely, may rely more on circuits related to the dorsal caudate), and changes in caudate connectivity could underlie working-memory dysfunction in PD (Simioni et al., 2017). Accordingly, because patients were assessed during the “on” phase, it is possible that negative relationships between the advantageous choices made in the IGT and the performance to the digit span backward may reflect the effect of dopaminergic medications that can enhance functions mediated by dorsal striatum and affect mental operations underlying to a greater extent the ventral striatum (MacDonald & Monchi, 2011).

Conversely, considering the GDT, the inhibition of irrelevant stimuli emerged as fundamental in making safe choices avoiding risky ones. Taking results together with what was stated earlier, it may be possible that inhibition can play an important role in hindering the impulse to choose risky options that are explicitly characterized by high rewards but also high losses and where possible consequences derived by each option are more predictable (compared with the IGT, where no information about the options was given at the beginning of the task). Moreover, working memory was not related to the GDT parameters, confirming that relationships with EFs can depend on the characteristic of the situation and of the task (in the GDT all data are always shown on the screen). Moreover, having all data explicitly available before making a choice can allow the decision-maker to rely on decisional processes that are more cognitively demanding than those used at least in the first blocks of the IGT (Colautti et al., 2022), thus involving in different ways both affective and cognitive components, according to the assumption that the two value-based conditions—namely, risk and uncertainty—can involve different neural circuits (Brand et al., 2004; Euteneuer et al., 2009; Xi et al., 2015).

Accordingly, to deepen the involvement of affective components in the IGT, it is worth mentioning the correlations which emerged between the IGT parameters and the difficulties in identifying and elaborating one’s feelings (as revealed by TAS scores). Such findings confirm that being aware of the affective components may support a functional integration of affective states into the decisional strategy to make optimal choices under conditions of uncertainty (but not of risk, as emerged from the absence of significant relationships between the TAS and the GDT parameters). In the GDT, the absence of high negative emotional states seems to be helpful to avoid choosing only the riskiest option, possibly supporting a lower involvement of the affective components under conditions of risk compared with uncertain conditions.

### Limitations

This study has some limitations. First, focusing the attention on the DLT, we stated that, conversely to what happened for the IGT or the GDT, feedback was not provided after each choice and it could contribute to explaining the absence of significant differences between PD and HC groups. In this way, feedback was provided at the end of each part of the task (i.e., every ten choices), so we cannot exclude that it could have biased the choices made in the following parts of the DLT.

Second, it is worth mentioning that, for practical issues, to check global cognitive functioning, we considered one of the most common instruments (Scheffels et al., 2020), i.e., the MMSE score (together with UPDRS Part I item 1), even if other, more sophisticated tools (such as the Montreal Cognitive Assessment) might be more sensitive for cognitive screening in PD (Nazem et al., 2009).

Moreover, comparative analyses of patients’ decisional performances during the pharmacological “on” versus “off” conditions would have offered a more comprehensive understanding of the possible effects of the dopaminergic medications on DM under value-based conditions. Furthermore, because of the lack of neurobiological data, the hypotheses about the relations between the behavioral results/cognitive tests and cerebral functioning are only speculative.

Additionally, it could be argued that the lack of a clinical group composed of patients with PD with ICDs may be a limitation of the present work, because ICDs might be a critical symptom of PD. Moreover, this prevented us from evaluating any differences, at least from a behavioral point of view, between individuals with and without ICDs. However, in this study we were mainly interested in deepening the decisional process in patients with PD without ICD for these main reasons. (1) Such clinical condition is more common in patients with PD (the prevalence of patients with ICDs was estimated to range from 20 to 45%; Weintraub & Claassen, 2017; Monaco et al., 2018); (2) It is not clear in the literature whether patients without ICDs can present decisional impairments or can be more prone to make risky and suboptimal choices compared with matched HCs, even if possible evidence in this way would offer precious information for developing more effective care pathways and preventing negative consequences in everyday life; and (3) Excluding patients with ICDs allowed us to avoid possible confounding influence in investigating decisional mechanisms and the relationships with the other considered variables (e.g., a recent meta-analysis supported the relationships between ICDs and dysfunctions in EFs, especially when set-shifting—which is pivotal for DM processes—was considered; Martini et al., 2018). Further studies adopting similar methodologies to investigate value-based decisional processes and possible relationships with dopaminergic medications, cognitive functioning, and individual differences also should consider patients with PD with ICD.

Last, considering the cross-sectional design, causal relationships cannot be drawn from the presented results. Further studies are needed in this way, also adopting longitudinal designs. However, despite these limitations, the study contributed to better understanding in a comprehensive way the cognitive mechanisms underlying DM processes in patients with PD, trying to find answers to some of the gaps to date present in literature.

## Conclusions

The present study delved into the DM mechanisms in patients with PD, both comparing the decisional performances between patients and HCs and analyzing the decisional performances to behavioral tasks in relation to the different dopamine medications, cognitive functioning, and individual differences. The results confirmed the presence of an attitude toward risky choices in patients with PD, both under conditions of uncertainty and risk, possibly explained by two similar but different mechanisms, in which dopaminergic medications can play a role in processing consequences. Moreover, findings about the DLT showed that patients with PD, similarly to HCs, perceived more difficult deciding in those situations in which specific information on the probability of occurrence of the possible consequences is lacking.

Findings confirmed that (1) EFs, and in particular inhibition and flexibility, can sustain an optimal decisional process avoiding risky decisions; (2) the involvement of EFs can change according to the characteristics of the tasks and the decisional conditions; and (3) it is important to keep the focus on the study of the effects of the differential dopaminergic medications when investigating value-based DM to deepen how they can differently influence the cognitive processes underlying DM.

### Clinical implications

From the present findings, several clinical implications can follow, especially considering that DM plays a pivotal role in patients’ quality of life and can influence their long-term goals, including adherence to therapy (Evens et al., 2016; Salvatore et al., 2021).

Specifically, identifying the abilities encompassed in EFs and involved in the DM process can support the design of cognitive programs to rehabilitate or enhance DM, also by fostering EFs. This becomes even more crucial when considering that patients with PD may develop selective cognitive difficulties, because the early stages of the disease and these impairments can worsen with the progression of the disease, being a risk factor for the development of dementia (Saredakis et al., 2019). Similarly, a focus on patients’ individual traits, such as the locus of control and the attitude for considering the long-term consequences of a decision, should be desirable in such programs. Future studies would be useful to understand whether and how enhancing EFs can be helpful to improve patients’ DM processes.

Moreover, further studies should delve into whether the presence of impairments in such decisional tasks could be an early indicator to detect those patients who are more prone to develop impairments in value-based DM or, in pathological cases, ICDs. This could prevent blatant behavioral impairments in everyday life, which can lead to detrimental consequences both for patients and their families.

Finally, focusing on patients’ higher perceived difficulty in making a decision when the probability of consequences is not declared, it could be useful to increase the quality of communication of clinicians toward patients providing, whether it is possible, such data to prevent patients’ risky choices. As well, when patients have to make a decision, it can be useful for practitioners to provide, where possible, the necessary time to weigh the possible options without increasing anxiety or encouraging impulsive decisions, ensuring that the patient has considered the short-term and long-term consequences for each option. In this way, increasing awareness of the possible variables involved in patients’ DM can support the design of effective and tailored clinical pathways to prolong the autonomy of patients.

## Supplementary information

Below is the link to the electronic supplementary material.

Supplementary file1 (DOCX 45 KB)

## Declarations

### Data availability

The data that support the findings of this study are available on request from the corresponding author. The data are not publicly available due to privacy or ethical restrictions. Materials concerning the Drawn Lots Task are available through the Open Science Framework at https://osf.io/cpjh4/?view_only=23bef8d21ee84b78a35d416e80803437.

### Open practices statement

Data or materials for the experiments reported are available on request from the corresponding author. None of the experiments was preregistered.
