# Supplementary material for: Deepening the decisional processes under value-based conditions in patients affected by Parkinson’s disease: A comparative study
Source: Cogn Affect Behav Neurosci. 2024 Sep 12;24(6):1167–85. doi: 10.3758/s13415-024-01211-x (PMC11525292; doi:10.3758/s13415-024-01211-x)
Supplement: Supplementary file 1 — Supplementary file1 (DOCX 45 KB) [file 13415_2024_1211_MOESM1_ESM.docx]

**Supplementary materials**

**Table S1**

*Correlation matrix using the Spearman’s rho between IGT netscores and dopaminergic medications.*

|  |  | **IGT_netscore_tot** | | **IGT_netscore_1-20** | | **IGT_netscore_21-40** | | **IGT_netscore_41-60** | | **IGT_netscore_61-80** | | **IGT_netscore_81-100** | |
| --- | --- | --- | --- | --- | --- | --- | --- | --- | --- | --- | --- | --- | --- |
| Ldopa_LEDD | Spearman rho | 0.027 |  | 0.197 |  | -0.194 |  | 0.127 |  | -0.125 |  | 0.216 |  |
|  | p | 0.867 |  | 0.212 |  | 0.218 |  | 0.424 |  | 0.430 |  | 0.170 |  |
| COMT_LEDD | Spearman rho | -0.034 |  | 0.281 |  | -0.185 |  | -0.023 |  | -0.029 |  | -0.023 |  |
|  | p | 0.832 |  | 0.072 |  | 0.241 |  | 0.884 |  | 0.855 |  | 0.884 |  |
| DA_LEDD | Spearman rho | -0.052 |  | 0.172 |  | -0.371 | * | -0.178 |  | 0.044 |  | 0.091 |  |
|  | p | 0.744 |  | 0.277 |  | 0.015 |  | 0.258 |  | 0.784 |  | 0.565 |  |
| MAO_LEDD | Spearman rho | 0.153 |  | -0.105 |  | 0.211 |  | 0.169 |  | 0.138 |  | 0.117 |  |
|  | p | 0.333 |  | 0.506 |  | 0.180 |  | 0.286 |  | 0.384 |  | 0.459 |  |
| LEDD_tot | Spearman rho | 0.030 |  | 0.207 |  | -0.286 |  | 0.056 |  | -0.005 |  | 0.218 |  |
|  | p | 0.849 |  | 0.189 |  | 0.066 |  | 0.726 |  | 0.977 |  | 0.166 |  |

COMT_LEDD: Levodopa equivalent daily dose of COMT inhibitors; DA_LEDD: Levodopa equivalent daily dose of dopamine agonists; Ldopa_LEDD: Levodopa equivalent daily dose of levodopa; LEDD_tot: Total levodopa equivalent daily dose; MAO_LEDD: Levodopa equivalent daily dose of MAO-B inhibitors.

* p < .05, ** p < .01, *** p < .001.

**Table S2**

*Correlation matrix using the Spearman’s rho between GDT parameters and dopaminergic medications.*

|  |  | **GDT_netscore** | | **GDT_risky_tot** | | **GDT_safe_tot** | | **GDT_1** | | **GDT_4** | |
| --- | --- | --- | --- | --- | --- | --- | --- | --- | --- | --- | --- |
| Ldopa_LEDD | Spearman rho | -0.407 | ** | 0.385 | * | -0.385 | * | 0.323 | * | -0.177 |  |
|  | p | 0.007 |  | 0.012 |  | 0.012 |  | 0.037 |  | 0.263 |  |
| COMT_LEDD | Spearman rho | -0.078 |  | 0.047 |  | -0.047 |  | 0.038 |  | 0.134 |  |
|  | p | 0.625 |  | 0.767 |  | 0.767 |  | 0.813 |  | 0.396 |  |
| DA_LEDD | Spearman rho | 0.199 |  | -0.269 |  | 0.269 |  | -0.114 |  | 0.008 |  |
|  | p | 0.206 |  | 0.085 |  | 0.085 |  | 0.473 |  | 0.959 |  |
| MAO_LEDD | Spearman rho | -0.087 |  | 0.152 |  | -0.152 |  | -0.001 |  | 0.057 |  |
|  | p | 0.585 |  | 0.336 |  | 0.336 |  | 0.997 |  | 0.718 |  |
| LEDD_tot | Spearman rho | -0.214 |  | 0.170 |  | -0.170 |  | 0.177 |  | -0.057 |  |
|  | p | 0.174 |  | 0.283 |  | 0.283 |  | 0.262 |  | 0.721 |  |

COMT_LEDD: Levodopa equivalent daily dose of COMT inhibitors; DA_LEDD: Levodopa equivalent daily dose of dopamine agonists; Ldopa_LEDD: Levodopa equivalent daily dose of levodopa; LEDD_tot: Total levodopa equivalent daily dose; MAO_LEDD: Levodopa equivalent daily dose of MAO-B inhibitors.

* p < .05, ** p < .01, *** p < .001.

**Table S3**

*Correlation matrix using the Spearman’s rho between IGT netscores and neuropsychological tests.*

|  |  | **IGT_netscore_tot** | | **IGT_netscore_1-20** | | **IGT_netscore_21-40** | | **IGT_netscore_41-60** | | **IGT_netscore_61-80** | | **IGT_netscore_81-100** | |
| --- | --- | --- | --- | --- | --- | --- | --- | --- | --- | --- | --- | --- | --- |
| Stroop_T | Spearman rho | 0.186 |  | -0.035 |  | 0.273 |  | 0.046 |  | 0.282 |  | 0.060 |  |
|  | p | 0.245 |  | 0.826 |  | 0.085 |  | 0.776 |  | 0.074 |  | 0.709 |  |
| Stroop_E | Spearman rho | 0.234 |  | 0.389 | * | 0.113 |  | 0.195 |  | 0.142 |  | 0.160 |  |
|  | p | 0.141 |  | 0.012 |  | 0.481 |  | 0.221 |  | 0.375 |  | 0.316 |  |
| FP | Spearman rho | 0.051 |  | 0.422 | ** | -0.292 |  | -0.165 |  | -0.008 |  | 0.095 |  |
|  | p | 0.747 |  | 0.005 |  | 0.060 |  | 0.297 |  | 0.962 |  | 0.550 |  |
| FS | Spearman rho | 0.086 |  | 0.267 |  | -0.253 |  | -0.093 |  | 0.028 |  | 0.116 |  |
|  | p | 0.590 |  | 0.088 |  | 0.106 |  | 0.558 |  | 0.861 |  | 0.465 |  |
| FA | Spearman rho | 0.309 | * | 0.083 |  | -0.122 |  | 0.099 |  | 0.266 |  | 0.286 |  |
|  | p | 0.047 |  | 0.601 |  | 0.443 |  | 0.535 |  | 0.088 |  | 0.067 |  |
| SI | Spearman rho | 0.347 | * | -0.230 |  | 0.137 |  | 0.263 |  | 0.378 | * | 0.278 |  |
|  | p | 0.024 |  | 0.143 |  | 0.386 |  | 0.093 |  | 0.014 |  | 0.075 |  |
| DF | Spearman rho | -0.110 |  | -0.093 |  | -0.011 |  | 0.100 |  | -0.138 |  | -0.132 |  |
|  | p | 0.487 |  | 0.559 |  | 0.946 |  | 0.528 |  | 0.384 |  | 0.406 |  |
| DB | Spearman rho | -0.393 | * | 0.088 |  | -0.388 | * | -0.326 | * | -0.345 | * | -0.369 | * |
|  | p | 0.010 |  | 0.578 |  | 0.011 |  | 0.035 |  | 0.025 |  | 0.016 |  |

DF: Digit Span Forward; DB: Digit Span Backward; FA: Alternate fluencies; FP: Phonemic fluencies; FS: Semantic fluencies; IGT: Iowa Gambling Task; SI: Shifting index; Stroop_E: Stroop test Errors; Stroop_T: Stroop test Time.

* p < .05, ** p < .01, *** p < .001.

**Table S4**

*Correlation matrix using the Spearman’s rho between GDT parameters and neuropsychological tests.*

|  |  | **GDT_netscore** | | **GDT_risky_tot** | | **GDT_safe_tot** | | **GDT_1** | | **GDT_4** | |
| --- | --- | --- | --- | --- | --- | --- | --- | --- | --- | --- | --- |
| Stroop_T | Spearman rho | -0.371 | * | 0.270 |  | -0.270 |  | 0.393 | * | -0.120 |  |
|  | p | 0.017 |  | 0.088 |  | 0.088 |  | 0.011 |  | 0.456 |  |
| Stroop_E | Spearman rho | 0.011 |  | -0.081 |  | 0.081 |  | 0.040 |  | 0.261 |  |
|  | p | 0.945 |  | 0.616 |  | 0.616 |  | 0.803 |  | 0.099 |  |
| FP | Spearman rho | 0.196 |  | -0.246 |  | 0.246 |  | -0.100 |  | 0.080 |  |
|  | p | 0.214 |  | 0.117 |  | 0.117 |  | 0.529 |  | 0.614 |  |
| FS | Spearman rho | -0.061 |  | 0.166 |  | -0.166 |  | 0.070 |  | -0.199 |  |
|  | p | 0.702 |  | 0.294 |  | 0.294 |  | 0.661 |  | 0.207 |  |
| FA | Spearman rho | 0.141 |  | -0.136 |  | 0.136 |  | -0.183 |  | -0.045 |  |
|  | p | 0.372 |  | 0.392 |  | 0.392 |  | 0.246 |  | 0.776 |  |
| SI | Spearman rho | 0.116 |  | -0.121 |  | 0.121 |  | -0.202 |  | -0.004 |  |
|  | p | 0.465 |  | 0.444 |  | 0.444 |  | 0.200 |  | 0.978 |  |
| DF | Spearman rho | 0.070 |  | 0.008 |  | -0.008 |  | -0.012 |  | 0.017 |  |
|  | p | 0.660 |  | 0.961 |  | 0.961 |  | 0.942 |  | 0.916 |  |
| DB | Spearman rho | -0.178 |  | 0.073 |  | -0.073 |  | 0.197 |  | -0.192 |  |
|  | p | 0.259 |  | 0.644 |  | 0.644 |  | 0.210 |  | 0.223 |  |

DF: Digit Span Forward; DB: Digit Span Backward; FA: Alternate fluencies; FP: Phonemic fluencies; FS: Semantic fluencies; GDT_1: Game of Dice Task – number of time the riskiest choice was made; GDT_4: Game of Dice Task – number of time the safest choice was made; GDT_risky_tot: Game of Dice Task – number of risky choices; GDT_safe_tot: Game of Dice Task – number of safe choices; SI: Shifting index; Stroop_E: Stroop test Errors; Stroop_T: Stroop test Time.

* p < .05, ** p < .01, *** p < .001.

**Table S5**

*Correlation matrix using the Spearman’s rho between IGT netscores and emotional and behavioral differences.*

|  |  | **IGT_netscore_tot** | | **IGT_netscore_1-20** | | **IGT_netscore_21-40** | | **IGT_netscore_41-60** | | **IGT_netscore_61-80** | | **IGT_netscore_81-100** | |
| --- | --- | --- | --- | --- | --- | --- | --- | --- | --- | --- | --- | --- | --- |
| DASS_depr | Spearman rho | -0.307 |  | 0.012 |  | -0.128 |  | -0.241 |  | -0.383 | * | -0.002 |  |
|  | p | 0.069 |  | 0.946 |  | 0.457 |  | 0.156 |  | 0.021 |  | 0.992 |  |
| DASS_anx | Spearman rho | -0.329 | * | -0.094 |  | -0.185 |  | -0.245 |  | -0.424 | ** | -0.017 |  |
|  | p | 0.043 |  | 0.574 |  | 0.267 |  | 0.139 |  | 0.008 |  | 0.920 |  |
| DASS_stress | Spearman rho | -0.126 |  | 0.135 |  | -0.010 |  | -0.140 |  | -0.215 |  | 0.105 |  |
|  | p | 0.452 |  | 0.418 |  | 0.955 |  | 0.403 |  | 0.195 |  | 0.532 |  |
| DASS_tot | Spearman rho | -0.248 |  | -0.029 |  | -0.098 |  | -0.171 |  | -0.324 |  | 0.051 |  |
|  | p | 0.145 |  | 0.866 |  | 0.570 |  | 0.318 |  | 0.054 |  | 0.766 |  |
| DII_FI | Spearman rho | 0.397 | * | 0.138 |  | 0.099 |  | 0.491 | ** | 0.392 | * | 0.109 |  |
|  | p | 0.014 |  | 0.408 |  | 0.553 |  | 0.002 |  | 0.015 |  | 0.516 |  |
| DII_DI | Spearman rho | -0.448 | ** | 0.155 |  | -0.306 |  | -0.547 | *** | -0.505 | ** | -0.077 |  |
|  | p | 0.005 |  | 0.353 |  | 0.062 |  | < .001 |  | 0.001 |  | 0.646 |  |
| CFC_fut | Spearman rho | 0.363 | * | -0.059 |  | 0.414 | ** | 0.383 | * | 0.139 |  | 0.237 |  |
|  | p | 0.025 |  | 0.724 |  | 0.010 |  | 0.018 |  | 0.406 |  | 0.152 |  |
| CFC_imm | Spearman rho | 0.016 |  | -0.027 |  | -0.181 |  | 0.107 |  | -0.019 |  | 0.104 |  |
|  | p | 0.925 |  | 0.872 |  | 0.278 |  | 0.524 |  | 0.908 |  | 0.533 |  |
| LCB_tot | Spearman rho | -0.290 |  | 0.165 |  | -0.271 |  | -0.163 |  | -0.387 | * | -0.078 |  |
|  | p | 0.077 |  | 0.322 |  | 0.100 |  | 0.328 |  | 0.016 |  | 0.641 |  |
| TAS_F1 | Spearman rho | -0.341 | * | 0.060 |  | -0.048 |  | -0.107 |  | -0.383 | * | -0.210 |  |
|  | p | 0.036 |  | 0.721 |  | 0.774 |  | 0.524 |  | 0.018 |  | 0.206 |  |
| TAS_F2 | Spearman rho | -0.143 |  | 0.111 |  | -0.053 |  | -0.183 |  | -0.261 |  | 0.035 |  |
|  | p | 0.399 |  | 0.512 |  | 0.755 |  | 0.279 |  | 0.119 |  | 0.838 |  |
| TAS_tot | Spearman rho | -0.328 | * | 0.143 |  | -0.253 |  | -0.274 |  | -0.379 | * | -0.080 |  |
|  | p | 0.047 |  | 0.399 |  | 0.131 |  | 0.100 |  | 0.021 |  | 0.636 |  |

CFC_fut: Consideration of Future Consequences Scale – future; CFC_imm: Consideration of Future Consequences Scale – immediate; DASS_anx: Depression Anxiety Stress Scale – anxiety; DASS_depr: Depression Anxiety Stress Scale – depression; DASS_stress: Depression Anxiety Stress Scale – stress; DASS_tot: Depression Anxiety Stress Scale – total score; DII_DI: Dickman Impulsivity Inventory – dysfunctional impulsivity; DII_FI: Dickman Impulsivity Inventory – functional impulsivity; LCB_tot: Locus of Control of Behavior; TAS_F1: Toronto Alexithymia Scale – difficulty in identifying feelings; TAS_F2: Toronto Alexithymia Scale – difficulty in describing feelings; TAS_tot: Toronto Alexithymia Scale – total score.

* p < .05, ** p < .01, *** p < .001.

**Table S6**

*Correlation matrix using the Spearman’s rho between GDT parameters and emotional and behavioral differences.*

|  |  | **GDT_netscore** | | **GDT_risky_tot** | | **GDT_safe_tot** | | **GDT_1** | | **GDT_4** | |
| --- | --- | --- | --- | --- | --- | --- | --- | --- | --- | --- | --- |
| DASS_depr | Spearman rho | -0.314 |  | 0.307 |  | -0.307 |  | 0.332 | * | -0.115 |  |
|  | p | 0.062 |  | 0.069 |  | 0.069 |  | 0.048 |  | 0.504 |  |
| DASS_anx | Spearman rho | -0.237 |  | 0.199 |  | -0.199 |  | 0.327 | * | -0.097 |  |
|  | p | 0.152 |  | 0.231 |  | 0.231 |  | 0.045 |  | 0.564 |  |
| DASS_stress | Spearman rho | -0.115 |  | 0.105 |  | -0.105 |  | 0.164 |  | 0.012 |  |
|  | p | 0.493 |  | 0.529 |  | 0.529 |  | 0.325 |  | 0.945 |  |
| DASS_tot | Spearman rho | -0.215 |  | 0.207 |  | -0.207 |  | 0.270 |  | -0.042 |  |
|  | p | 0.209 |  | 0.227 |  | 0.227 |  | 0.111 |  | 0.809 |  |
| DII_FI | Spearman rho | -0.179 |  | 0.247 |  | -0.247 |  | 0.031 |  | -0.047 |  |
|  | p | 0.282 |  | 0.135 |  | 0.135 |  | 0.852 |  | 0.779 |  |
| DII_DI | Spearman rho | -0.243 |  | 0.149 |  | -0.149 |  | 0.231 |  | -0.220 |  |
|  | p | 0.141 |  | 0.372 |  | 0.372 |  | 0.163 |  | 0.184 |  |
| CFC_fut | Spearman rho | 0.194 |  | -0.174 |  | 0.174 |  | -0.229 |  | 0.306 |  |
|  | p | 0.242 |  | 0.295 |  | 0.295 |  | 0.167 |  | 0.061 |  |
| CFC_imm | Spearman rho | -0.187 |  | 0.284 |  | -0.284 |  | 0.270 |  | -0.251 |  |
|  | p | 0.261 |  | 0.084 |  | 0.084 |  | 0.101 |  | 0.129 |  |
| LCB_tot | Spearman rho | -0.211 |  | 0.175 |  | -0.175 |  | 0.255 |  | -0.238 |  |
|  | p | 0.204 |  | 0.293 |  | 0.293 |  | 0.122 |  | 0.150 |  |
| TAS_F1 | Spearman rho | -0.069 |  | 0.006 |  | -0.006 |  | 0.197 |  | 0.233 |  |
|  | p | 0.683 |  | 0.969 |  | 0.969 |  | 0.236 |  | 0.158 |  |
| TAS_F2 | Spearman rho | -0.107 |  | 0.074 |  | -0.074 |  | 0.156 |  | 0.093 |  |
|  | p | 0.528 |  | 0.665 |  | 0.665 |  | 0.357 |  | 0.584 |  |
| TAS_tot | Spearman rho | -0.121 |  | 0.016 |  | -0.016 |  | 0.228 |  | 0.109 |  |
|  | p | 0.477 |  | 0.923 |  | 0.923 |  | 0.175 |  | 0.521 |  |

CFC_fut: Consideration of Future Consequences Scale – future; CFC_imm: Consideration of Future Consequences Scale – immediate; DASS_anx: Depression Anxiety Stress Scale – anxiety; DASS_depr: Depression Anxiety Stress Scale – depression; DASS_stress: Depression Anxiety Stress Scale – stress; DASS_tot: Depression Anxiety Stress Scale – total score; DII_DI: Dickman Impulsivity Inventory – dysfunctional impulsivity; DII_FI: Dickman Impulsivity Inventory – functional impulsivity; LCB_tot: Locus of Control of Behavior; TAS_F1: Toronto Alexithymia Scale – difficulty in identifying feelings; TAS_F2: Toronto Alexithymia Scale – difficulty in describing feelings; TAS_tot: Toronto Alexithymia Scale – total score.

* p < .05, ** p < .01, *** p < .001.
